# Osmotin attenuates amyloid beta-induced memory impairment, tau phosphorylation and neurodegeneration in the mouse hippocampus

**DOI:** 10.1038/srep11708

**Published:** 2015-06-29

**Authors:** Tahir Ali, Gwang Ho Yoon, Shahid Ali Shah, Hae Young Lee, Myeong Ok Kim

**Affiliations:** 1 Department of Biology and Applied Life Science (BK 21), College of Natural Sciences, (RINS), Gyeongsang National University, Jinju, 660-701, Republic of Korea

## Abstract

The pathological hallmarks of Alzheimer’s disease (AD) include amyloid beta (Aβ) accumulation, neurofibrillary tangle formation, synaptic dysfunction and neuronal loss. In this study, we investigated the neuroprotection of novel osmotin, a plant protein extracted from *Nicotiana tabacum* that has been considered to be a homolog of mammalian adiponectin. Here, we observed that treatment with osmotin (15 μg/g, intraperitoneally, 4 hr) at 3 and 40 days post-intracerebroventricular injection of Aβ_1-42_ significantly ameliorated Aβ_1-42_-induced memory impairment in mice. These results revealed that osmotin reverses Aβ_1-42_ injection-induced synaptic deficits, Aβ accumulation and BACE-1 expression. Treatment with osmotin also alleviated the Aβ_1-42_-induced hyperphosphorylation of the tau protein at serine 413 through the regulation of the aberrant phosphorylation of p-PI3K, p-Akt (serine 473) and p-GSK3β (serine 9). Moreover, our western blots and immunohistochemical results indicated that osmotin prevented Aβ_1-42_-induced apoptosis and neurodegeneration in the Aβ_1-42_-treated mice. Furthermore, osmotin attenuated Aβ_1-42_-induced neurotoxicity *in vitro*.

To our knowledge, this study is the first to investigate the neuroprotective effect of a novel osmotin against Aβ_1-42_-induced neurotoxicity. Our results demonstrated that this ubiquitous plant protein could potentially serve as a novel, promising, and accessible neuroprotective agent against progressive neurodegenerative diseases such as AD.

Alzheimer’s disease (AD) is an age-related neurodegenerative disorder. AD is the most prevalent cause of dementia in the elderly and is characterized by the progressive dysfunction of memory and higher cognitive functions. The neuropathological hallmarks of AD include senile plaques, neurofibrillary tangles, synaptic dysfunction and neuronal loss in the brain. Senile plaques are extracellular aggregates consisting of amyloid beta (Aβ) peptides, and neurofibrillary tangles are composed of hyperphosphorylated tau protein^1^,^2^.

Aβ peptides are primarily generated from the cleavage of the transmembrane glycoprotein amyloid precursor protein (APP) by the enzymes β-secretase and γ-secretase^3^. Aβ plaque formation is a major pathological event in the brains of AD patients and results in memory and cognitive dysfunction. Aβ acts as a neurotoxin by initiating a group of biochemical cascades that ultimately lead to synaptotoxicity and neurodegeneration^4^. In addition to Aβ_1-42_ accumulation, tau hyperphosphorylation is an important pathological hallmark of AD. The increased aggregation of phosphorylated tau protein decreases microtubule binding, leading to axonal transport dysfunction and neuronal loss^5^. Nevertheless, the molecular mechanisms underlying Aβ accumulation, tau phosphorylation, synaptic loss and neurodegeneration remain unknown.

Advancements in the prevention and treatment of neurodegenerative diseases, such as AD, have been made using natural products; therefore, plant-derived compounds are promising for the treatment of these conditions^6^. Osmotin is a plant protein that belongs to the pathogenesis-related (PR)-5 family of the plant defense system, which includes the sweet-tasting thaumatin protein^7^. Osmotin is a homolog of the mammalian adiponectin hormone (based on both structural and functional similarity). An adiponectin agonist for biological activities, osmotin mimics the anti-inflammatory activity of adiponectin in murine models of colitis^8^,^9^. Several studies have reported that adiponectin acts as a neuroprotective agent against various neurotoxic insults, e.g., kainic acid-induced excitotoxicity in rat hippocampal neurons and 1-methyl-4-phenylpyridinium ion (MPP^+^)-induced apoptosis in human SH-SY5Y neuroblastoma cells^10^,^11^. Prior to 2012, a study by Une, K. *et al.* (2011) reported that a high circulating level of adiponectin leads to cognitive impairments^12^. Moreover, Bigalke, B. *et al.* (2011) and Gu, Y*. et al.* (2010) did not observe any significant difference or correlation in circulating adiponectin levels between AD patients and healthy subjects^13^,^14^. However, after 2011, Chan, H. K. *et al.* (2012) found that adiponectin protects against Aβ-induced neurotoxicity in SH-SY5Y cells^15^, Diniz, B.S. *et al.* (2012) observed a reduced serum adiponectin level in elderly patients with major depression^16^, Teixeira, A. L. *et al.* (2013) reported an association between low levels of adiponectin and mild cognitive impairment in AD patients^17^, and recently, Miao, J. *et al.* (2013) revealed that the overexpression of adiponectin enhanced the behavioral performance of aged mice to a greater extent than young mice^18^. Furthermore, Song, J. and Lee, J.E. (2013) reported that adiponectin is a novel target for the treatment of AD^19^. In our group, Shah, S.A. *et al.* (2014) and Naseer, M.I. *et al.* (2014) recently demonstrated the neuroprotective effect of osmotin against glutamate- and ethanol-induced apoptosis and neurodegeneration in the postnatal rat brain^20^,^21^. We therefore investigated whether osmotin, a homolog of the mammalian adiponectin hormone, exerts a neuroprotective effect against Aβ_1-42_-induced memory impairment, synaptotoxicity, tau hyperphosphorylation and hippocampal neuronal degeneration in AD.

## Results

### Osmotin treatment ameliorates Aβ_1-42_-induced memory impairment

**Figure 1:**
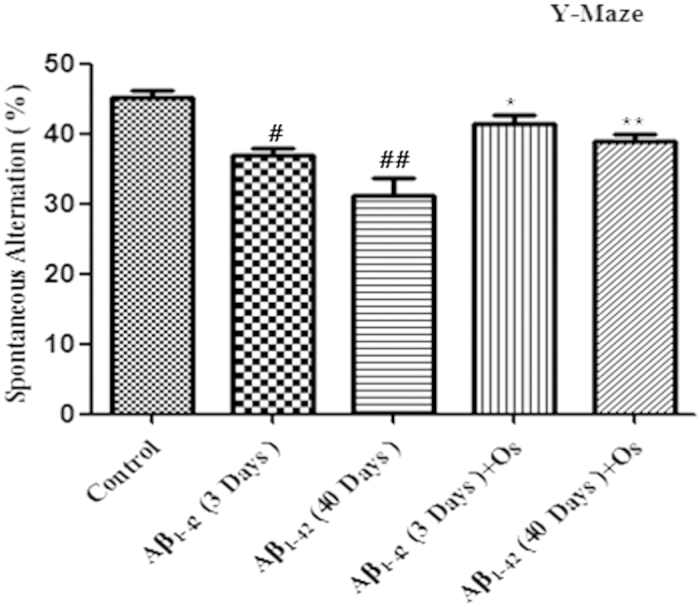
Effect of osmotin on spontaneous alternation behavior. The mice were treated with Aβ_1-42_ (3 μl/mouse, i.c.v.) or vehicle (control) and maintained for 3 or 40 days, represented by Aβ_1-42_ (3 days), Aβ_1-42_ (40 days) and control. Osmotin (15 μg/g, i.p., 4 hr) was administered to the mice on days 3 and 40 post-injection of Aβ_1-42_, represented by Aβ_1-42_ (3 days) +Os and Aβ_1-42_ (40 days) +Os, respectively. The spontaneous alternation behavior percentages were measured for 8 min using the Y-maze task in the respective groups after 4 hr of osmotin and saline administration The columns represent the means ± SEM; n = 15 for each experimental group. #significantly different from the vehicle-treated control mice; *significantly different from the Aβ_1-42_-treated mice.

To evaluate the effects of osmotin on memory impairment induced by Aβ_1-42_ injection, we evaluated the spontaneous alternation behavior of mice (n = 15/group) after 4 hr of osmotin and saline injection at 3 and 40 days post-injection of Aβ_1-42_ using a Y-maze test. Spontaneous alternation behavior is a measure of spatial working memory, which is a form of short-term memory. After a single injection of Aβ_1-42_, the percentage of spontaneous alternation behavior was significantly reduced after 3 and 40 days in the Aβ_1-42_-treated mice compared with the control mice. We subjected the control mice to the Y-maze at 3 days and at 40 days, and the spontaneous alternation behavior was the same in both groups. Therefore, we used the 40-day control group for behavioral and further molecular analyses. The results suggested that Aβ_1-42_ injection induced memory dysfunction. Treatment with osmotin (15 μg/g, i.p., 4 hr) significantly increased spontaneous alternation behavior at 3 and 40 days post-Aβ_1-42_ injection compared with mice injected with Aβ_1-42_ alone (p < 0.05, p < 0.01, Fig. 1), indicating that osmotin ameliorated Aβ_1-42_-induced memory impairment.

### Osmotin treatment alleviated Aβ_1-42_-induced synaptotoxicity

To assess synaptic integrity after Aβ_1-42_ treatment, we quantified the expression of presynaptic vesicle membrane proteins [synaptophysin and synaptosomal-associated protein 25 (SNAP-25)] and postsynaptic markers [post-synaptic density protein 95 (PSD95) and α-amino-3-hydroxy-5-methylisoxazol-4-propionic acid (AMPA) receptors (AMPARs)].

**Figure 2:**
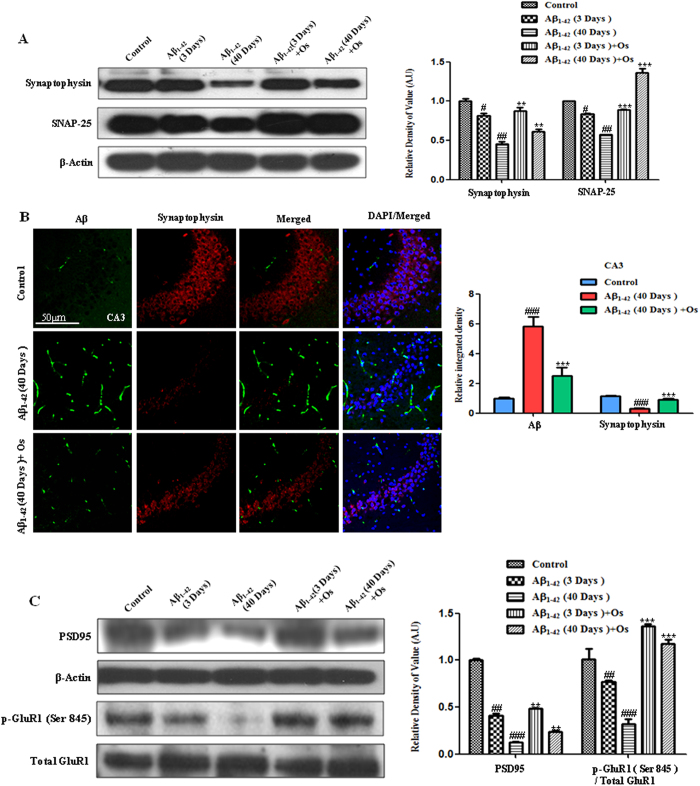
Osmotin reduced Aβ_1-42_-induced synaptotoxicity. (**A**) Western blot analysis of the mouse hippocampus using anti-synaptophysin and anti-SNAP-25 antibodies. The cropped bands were quantified using Sigma Gel software, and the differences are represented in the histogram. An anti-β-actin antibody was used as a loading control. The band density values are expressed in arbitrary units (A.U.) as the means ± SEM for the indicated proteins (n = 10 mice/group). (**B**) Representative images showing the results of immunofluorescence reactivity for Aβ (D-11) (FITC-labeled, green) and synaptophysin (TRITC-labeled, red). The 40-day post-Aβ_1-42_-treated mice exhibited decreased synaptic strength based on a reduction in synaptophysin immunoreactivity compared with the control mice. Osmotin treatment prevented the Aβ_1-42_-induced reduction in immunofluorescence reactivity for synaptophysin. #significantly different from the vehicle-treated control mice; *significantly different from the Aβ_1-42_-treated mice. n = 5 mice/group, n = 3 experiment. Magnification 40x; scale bar = 50 μm. (**C**) Western blot analysis of the mouse hippocampus using anti-p-GluR1 (Ser845), anti-total GluR1 and anti-PSD95 antibodies. The cropped bands were quantified using Sigma Gel software, and the differences are represented in the histogram. An anti-β-actin antibody was used as a loading control. The band density values are expressed in A.U. as the means ± SEM for the indicated proteins (n = 10 mice/group). #significantly different from the vehicle-treated control mice; *significantly different from the Aβ_1-42_-treated mice.

A western blot analysis showed a significant reduction in synaptophysin and SNAP-25 levels in Aβ_1-42_-treated mice after 3 days and 40 days post-Aβ_1-42_ injection compared with the control, indicating the induction of synaptic dysfunction (Fig. 2A). Osmotin treatment (15 μg/g, i.p., 4 hr) significantly increased synaptophysin (p < 0.01) and SNAP-25 (p < 0.001) expression after 3 and 40 days post-Aβ_1-42_ injection compared with Aβ_1-42_ alone (Fig. 2A).

The brain tissue was also histologically examined for synaptophysin expression via immunofluorescence. Representative images (Fig. 2B) showed that (at 40 days) post-Aβ_1-42_ injection reduced the immunofluorescence reactivity for synaptophysin (TRITC-labeled, red) and increased that for Aβ (FITC-labeled, green) in the CA3 region of the hippocampus compared with the control treatment. Osmotin treatment significantly increased the immunofluorescence reactivity for synaptophysin and decreased that for Aβ (p < 0.001, Fig. 2B).

The western blot results revealed a significant decrease in the PSD95 level in the Aβ_1-42_-treated groups at both 3 and 40 days compared with the control group. However, the magnitude of this effect after 40 days was greater than that after 3 days. Treatment with osmotin reduced this effect for Aβ_1-42_ and significantly increased the level of PSD95 at both 3 and 40 days post-injection compared with Aβ_1-42_ treatment alone (p < 0.01, Fig. 2C).

Aβ_1-42_-induced synaptic dysfunction has been associated with the alternation of AMPARs, notably the phosphorylation of the AMPAR 1 subunit (GluR1) at Ser845, which plays an important role in the trafficking of postsynaptic glutamate receptors^22^. Therefore, we examined the phosphorylation of GluR1 at Ser845. The results revealed that the level of p-GluR1 at Ser845 was significantly reduced after both 3 and 40 days in the Aβ_1-42_-treated mice compared with the control mice. Similarly to the PDS95 expression, the magnitude of the effect of Aβ_1-42_ treatment after 40 days was greater than that after 3 days. Treatment with osmotin significantly increased the levels of p-GluR1 at Ser845 at 3 and 40 days post-injection compared with Aβ_1-42_ injection alone (p < 0.001, Fig. 2C).

### Osmotin attenuated Aβ accumulation and β-site APP-cleaving enzyme-1 (BACE-1) expression

**Figure 3:**
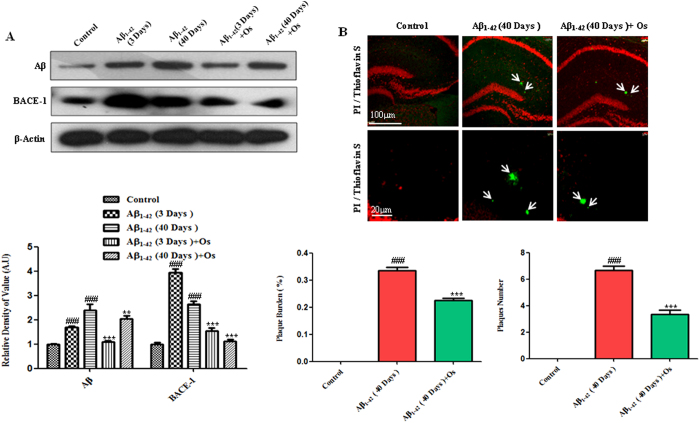
Osmotin attenuated the expression levels of Aβ and BACE-1. (**A**) Western blot analysis of Aβ (D-11) and BACE-1 expression in the mouse hippocampus. The cropped bands were quantified using Sigma Gel software, and the differences are represented in the graphs. β-actin was used as a loading control. The density values are expressed in A.U. as the means ± SEM for the indicated proteins (n = 10 mice/group). (**B**) Thioflavin S staining demonstrating the formation of Aβ plaques at 40 days post-Aβ_1-42_ injection. Treatment with osmotin significantly reduced the plaque number and burden (%) compared with Aβ_1-42_ treatment alone. n = 5 mice/group, n = 3 experiment. Magnification 10x and 40x. Scale bar = 100 μm and 20 μm. #significantly different from the vehicle-treated control mice; *significantly different from the Aβ_1-42_-treated mice.

To determine whether Aβ_1-42_ injection promoted Aβ accumulation, we performed western blot analysis. The results showed that the levels of Aβ were significantly higher in the Aβ_1-42_-treated mice at 3 and 40 days post-injection than in the control mice. Notably, the level of Aβ was higher after 40 days than after 3 days. Osmotin (15 μg/g, i.p., 4 hr) administration ameliorated this effect of Aβ_1-42_ due to a significant reduction in Aβ accumulation after both 3 and 40 days compared with Aβ_1-42_ treatment alone (p < 0.001 and p < 0.01, Fig. 3A).

To examine plaque formation after 40 days of Aβ_1-42_ injection, we performed thioflavin S staining. In the Aβ_1-42_-treated mice, the number of plaques and the plaque burden (%) were determined; no plaque formation was observed in the control mice. Treatment with osmotin significantly decreased the number of plaques and the plaque burden (%) compared with Aβ_1-42_ treatment alone (p <  0.001, Fig. 3B).

Further we also analyzed the immunofluorescence of Aβ (6E10) in the experimental mice of 40 days groups. Osmotin treatment significantly reduced the immunofluorescence reactivity of Aβ (6E10) in the CA3 (p <  0.001) and CA1 (p <  0.001) region of hippocampus in the Aβ_1-42_-treated group (Supp. Fig. 1).

We examined the expression of BACE-1 after Aβ_1-42_ injection, and the western blot analysis results showed that Aβ_1-42_ treatment significantly increased BACE-1 expression after both 3 and 40 days compared with the control treatment. Interestingly, at 3 days, BACE-1 expression was higher than that at 40 days post-Aβ_1-42_ treatment, suggesting that either BACE-1 is expressed independently of Aβ_1-42_ or that BACE-1 is involved in a potential negative feedback mechanism. Moreover, osmotin significantly decreased the expression of active BACE-1 after 3 and 40 days compared with Aβ_1-42_ alone (p < 0.001, Fig. 3A).

### Osmotin treatment prevents the Aβ_1-42_-induced hyperphosphorylation of tau through the regulation of PI3K/Akt/GSK-3β signaling

Considering the protective effect of osmotin on synaptophysin toxicity, Aβ accumulation and BACE-1 expression, we examined the effects of osmotin on tau phosphorylation in Aβ_1-42_-treated mice. The dysregulation of the PI3K/Akt/GSK3β signaling pathway, which affects tau hyperphosphorylation, has been associated with the Aβ model of AD^23^.

**Figure 4:**
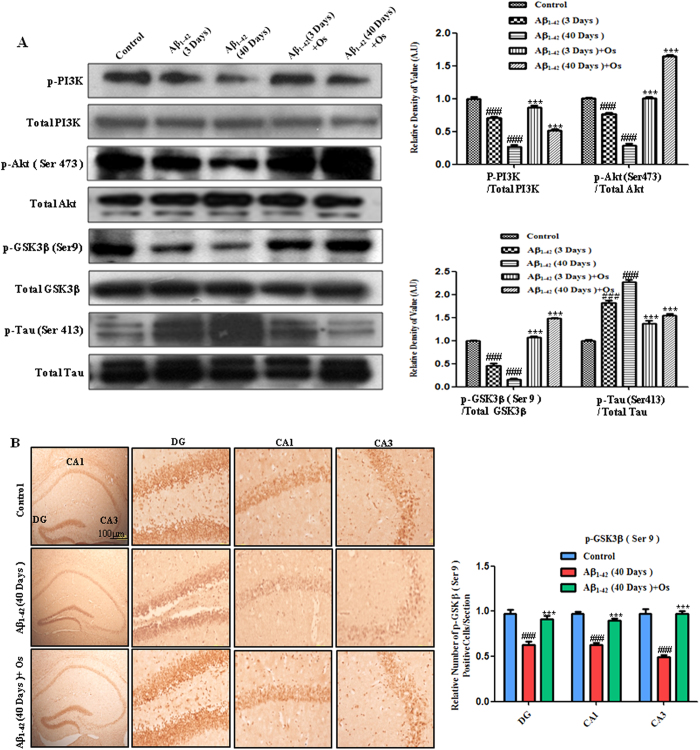
Osmotin treatment prevents Aβ-induced tau hyperphosphorylation via the regulation of the PI3K/Akt/GSK-3β signaling pathway. (**A**) Western blot analysis of the mouse hippocampus using anti-p-PI3K, anti-total PI3K, anti-p-Akt (Ser473), anti-total Akt, anti-p-GSK-3β (Ser9), anti-total GSK3β, anti-p-Tau (Ser413) and anti-total tau antibodies. The cropped bands were quantified using Sigma Gel software, and the differences are represented by the histogram. An anti-β-actin antibody was used as a loading control. The band density values are expressed in A.U. as the means ± SEM for the indicated proteins (n = 10 mice/group). #significantly different from the vehicle-treated control mice; *significantly different from the Aβ_1-42_-treated mice. (**B**) Immunohistochemistry for p-GSK3β (Ser9) showing that p-GSK3β (Ser9) immunoreactivity was decreased in the 40-day post-Aβ_1-42_-treated mice. Treatment with osmotin significantly increased the expression of p-GSK3β (Ser9) compared with Aβ_1-42_ treatment alone in the DG, CA1 and CA3 regions of the hippocampus. n = 5 mice/group, n = 3 experiment. Scale bar = 100 μm. #significantly different from the vehicle-treated control mice; *significantly different from the Aβ_1-42_-treated mice.

Western blot analysis revealed that the phosphorylated phosphatidylinositol 3-kinase (p-PI3K) was significantly reduced in the Aβ_1-42_-treated mice after both 3 and 40 days compared with the control mice. However, the magnitude of this effect after 40 days was greater than that after 3 days. The administration of osmotin (15 μg/g, i.p., 4 hr) significantly elevated the levels of p-PI3K after both 3 and 40 days compared with Aβ_1-42_ injection alone (p < 0.001, Fig. 4A).

Western blot analysis revealed that the phosphorylation of Akt at Ser473 was significantly reduced in the Aβ_1-42_-treated mice after both 3 and 40 days compared with the control mice. Similar to the results for p-PI3K, the magnitude of this effect after 40 days was greater than that after 3 days. The administration of osmotin (15 μg/g, i.p., 4 hr) significantly elevated the levels of p-Akt (Ser473) after both 3 and 40 days compared with Aβ_1-42_ injection alone (p < 0.001, Fig. 4A).

Glycogen synthase kinase 3 beta (GSK-3β) activity is inhibited by its phosphorylation at Ser9 via the phosphorylation of Akt^24^. The western blot results showed that the phosphorylation of GSK3β at Ser9 was significantly reduced at both 3 and 40 days after Aβ treatment compared with the control treatment. Osmotin treatment at both 3 and 40 days significantly increased the phosphorylation of GSK3β at Ser9 compared with Aβ_1-42_ treatment alone (p < 0.001, Fig. 4A). Immunohistochemical analysis revealed that the expression of p-GSK3β (Ser9) was decreased in the DG, CA1 and CA3 regions of the hippocampus after the 40-day Aβ_1-42_-treated mice compared with the control mice. Treatment with osmotin reversed this effect of Aβ_1-42_, and osmotin treatment significantly increased the expression of p-GSK3β (Ser9) compared with Aβ_1-42_ treatment alone in the DG, CA1 and CA3 regions of the hippocampus (p < 0.001, Fig. 4B).

We investigated the phosphorylation of the tau protein (p-tau) at serine 413 (Ser 413) in the control and Aβ_1-42_-treated mice via western blot analysis. Treatment with Aβ_1-42_ increased the level of p-tau (Ser413) after both 3 and 40 days compared with the control treatment. Osmotin treatment significantly attenuated the Aβ_1-42_-induced hyperphosphorylation of tau at Ser 413 after both 3 and 40 days compared with Aβ_1-42_ treatment alone (p < 0.001, Fig. 4A).

**Figure 5:**
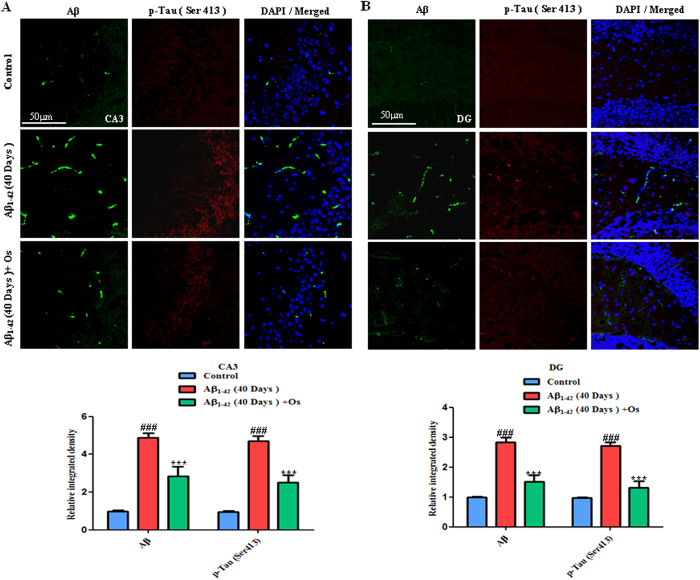
(**A**,**B**) Representative images showing immunofluorescence using the anti-Aβ (D-11) (FITC-labeled, green) and anti-p-tau (Ser413) (TRITC-labeled, red) antibodies. The mice treated 40 days post-Aβ_1-42_exhibited increased Aβ (green FITC-labeled) and p-Tau (Ser413) (red TRITC-labeled) immunofluorescence reactivity in the CA3 and DG regions of the hippocampus. Treatment with osmotin ameliorated the effects of Aβ_1-42_ and significantly decreased the immunoreactivity for p-tau (Ser413) and Aβ (D-11). n = 5 mice/group, n = 3 experiment. Magnification 40x; scale bar = 50 μm. #significantly different from the vehicle-treated control mice; *significantly different from the Aβ_1-42_-treated mice.

Furthermore, we examined the p-tau (Ser413) and Aβ levels via immunofluorescence. Consistent with the western blot analysis results, representative images (Fig. 5A,B) showed that at 40 days, post-Aβ_1-42_ injection significantly increased the Aβ (FITC-labeled, green) and p-tau immunofluorescence reactivity at Ser 413 (TRITC-labeled, red) compared with the control mice in the CA3 and DG regions of the hippocampus. Treatment with osmotin significantly reduced these effects of Aβ_1-42_ in the CA3 and DG regions of the hippocampus (p < 0.001; Fig. 5A,B).

### Osmotin prevents the apoptosis and neurodegeneration induced by Aβ_1-42_

Previous studies determined that the apoptotic activity of Aβ_1-42_ plays a critical role in neurodegeneration in AD^25^. Studies have shown that the PI3K/Akt/GSK3β neuroprotective and survival pathway is directly affected by Aβ exposure and that the activity of this pathway is impaired in the AD brain^26^. Adiponectin has been reported to activate various survival pathways. One important survival pathway is the PI3K/Akt pathway, which is activated by adiponectin and prevents apoptosis^27^. In plants, osmotin acts as a pro-apoptotic factor and is expressed in many fruits, seeds and vegetables, such as grapes, oats and tomatoes^28^. In our animal model of this study, osmotin appeared to protect against Aβ_1-42_-induced apoptosis. Recently, our group, Shah, S.A. *et al.* (2014) and Naseer, M.I. *et al.* (2014) demonstrated the neuroprotective effect of osmotin against glutamate- and ethanol-induced apoptosis and neurodegeneration in the postnatal rat brain^20^,^21^. Aβ_1-42_ activates the expression of the pro-apoptotic p53 protein, which mediates the activation of caspases in hippocampal neurons^29^. We performed a western blot analysis to determine whether osmotin suppresses neuronal apoptosis via p53-mediated caspase-associated apoptotic pathways in the hippocampus of Aβ_1-42_-treated mice.

**Figure 6:**
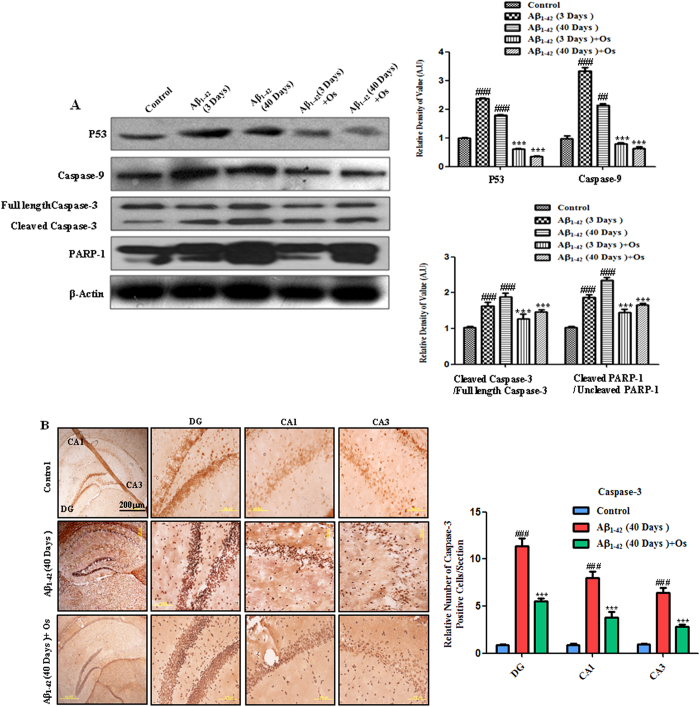
Osmotin prevents Aβ_1-42_-induced apoptosis and neurodegeneration. (**A**) Western blot analysis of the mouse hippocampus using anti-p53, anti-caspase-9, anti-cleaved caspase-3 and anti-PARP-1 antibodies. The cropped bands were quantified using Sigma Gel software, and the differences are represented by the histogram. An anti-β-actin antibody was used as a loading control. The band density values are expressed in A.U. as the means ± SEM for the indicated hippocampal proteins (n = 10 mice/group). #significantly different from the vehicle-treated control mice; *significantly different from the Aβ_1-42_-treated mice. (**B**) The cells that were immunoreactive to the anti-activated caspase-3 antibody were examined in the DG, CA3 and CA1 regions of the hippocampus of the mice treated 40 days post-Aβ_1-42_. The number of caspase-3-positive cells was increased in the Aβ_1-42_-treated mice compared with the control mice. Treatment with osmotin significantly ameliorated the Aβ-induced increase in the number of caspase-3-positive cells. n = 5 mice/group, n = 3 experiment. Scale bar = 200 μm. #significantly different from the vehicle-treated control mice; *significantly different from the Aβ_1-42_-treated mice.

Aβ_1-42_ significantly increased the level of p53 in the mice treated with Aβ_1-42_ after both 3 and 40 days compared with the control mice. The administration of osmotin (15 μg/g, i.p., 4 hr) to the mice treated with Aβ_1-42_ significantly decreased the level of p53 after both 3 and 40 days compared with Aβ_1-42_ alone (p < 0.001, Fig. 6A).

Caspases are serine-aspartyl proteases that are involved in the initiation and execution of apoptosis^30^. Caspase-9 is an initiator caspase. Western blot analysis revealed increased activation of caspase-9 at both 3 and 40 days after Aβ_1-42_ treatment compared with the control treatment. Treatment with osmotin significantly decreased Aβ_1-42_-induced caspase-9 activation in the hippocampus after both 3 and 40 days compared with Aβ_1-42_ treatment alone (p < 0.001, Fig. 6A).

Caspase-3 is an executor caspase that acts downstream of other caspases, such as caspase-9. We investigated the levels of caspase-3 in response to Aβ_1-42_ treatment via western blot analysis to determine whether osmotin reduces the Aβ_1-42_-induced elevation in the expression of active caspase-3. Our results showed that the level of activated caspase-3 was higher in the Aβ_1-42_-treated mice after both 3 and 40 days compared with the control mice. Treatment with osmotin ameliorated the Aβ_1-42_-induced upregulation of active caspase-3 and significantly decreased the level of caspase-3 after both 3 and 40 days compared with Aβ_1-42_ treatment alone (p < 0.001, Fig. 6A).

Activated caspase-3 expression was also examined via immunohistochemical analysis. The number of active caspase-3-positive cells was significantly higher in the DG, CA3 and CA1 regions of the hippocampus after 40 days in the Aβ_1-42_-injection mice compared with the control mice (Fig. 6B). After osmotin administration, the number of active caspase-3-positive cells was significantly decreased compared with Aβ_1-42_ treatment alone in the DG, CA1 and CA3 regions of the hippocampus (p < 0.001, Fig. 6B).

Poly (ADP-ribose) polymerase-1 (PARP-1) is involved in DNA repair, and the hyperactivation of PARP-1 in response to an excitotoxic insult induces neurodegeneration^31^. Aβ peptide increases the activity of PARP-1 in the hippocampus of adult rats^32^. PARP-1 is cleaved following the activation of caspase-3, subsequently resulting in apoptosis and, ultimately, neuronal death^33^. Western blot analysis revealed that PARP-1 cleavage in the hippocampus of the Aβ_1-42_-treated mice reflected increased caspase-3 activity, which occurs during apoptosis and neurodegeneration. The level of cleaved PARP-1 was significantly increased in the mice treated with Aβ_1-42_ after both 3 and 40 days compared with the control mice. Treatment with osmotin significantly reduced PARP-1 cleavage in the hippocampus compared with Aβ_1-42_ treatment alone (p < 0.001, Fig. 6A).

**Figure 7:**
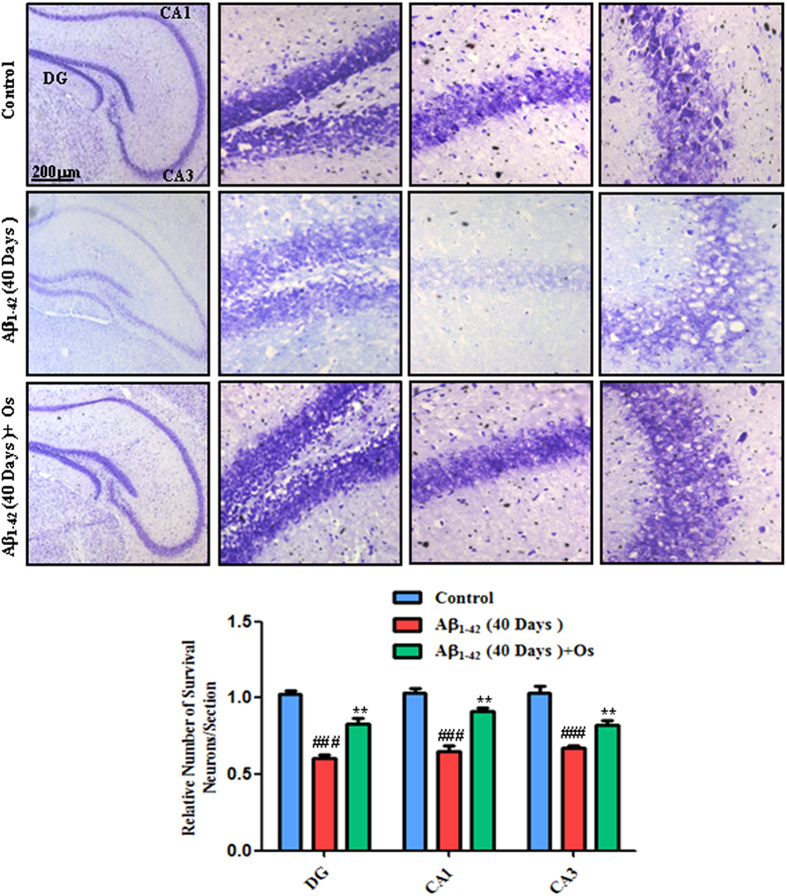
Representative photomicrograph of Nissl staining in the DG, CA3 and CA1 regions of the mouse hippocampus. n = 5 mice/group, n = 3 experiment. Scale bar = 200 μm. #significantly different from the vehicle-treated control mice; *significantly different from the Aβ_1-42_-treated mice.

Furthermore, Nissl staining was performed to investigate the extent of neuronal death in the hippocampus induced by Aβ_1-42_ injection in the 40 days group and to assess the protection conferred by osmotin administration to the Aβ_1-42_-treated mice. The number of survival neurons in the DG, CA3 and CA1 regions was significantly reduced in the Aβ_1-42_-treated mice compared with the control mice. Treatment with osmotin blocked this effect of Aβ_1-42_ and significantly increased the number of survival neurons compared with Aβ_1-42_ treatment alone (p < 0.01, Fig. 7).

### Effect of osmotin against Aβ_1-42_-induced neurotoxicity *in vitro*

**Figure 8:**
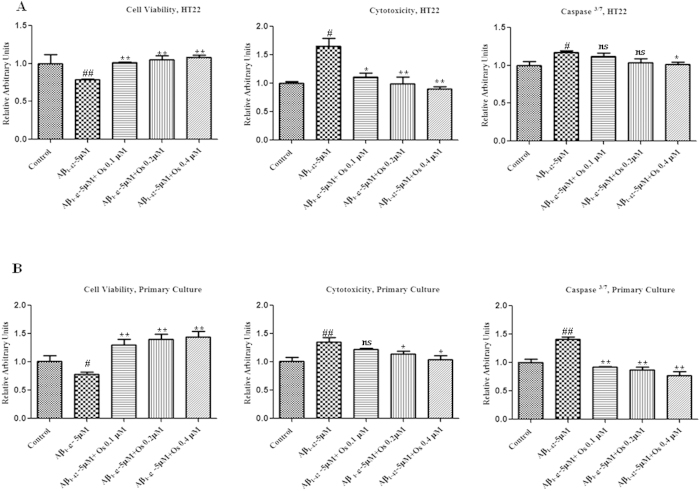
Osmotin attenuated the deleterious effects of Aβ_1-42_ *in vitro*. (**A**). The ApoTox-Glo^TM^ assay in neuronal HT22 cells. Cell viability was decreased but cytotoxicity and caspase-^3/7^ activation were increased after treatment with Aβ_1-42_ (5 μM) compared with the control treatment. Treatment with osmotin at three different concentrations (0.1, 0.2, or 0.4 μM) significantly reduced the effects of Aβ_1-42_, thereby increasing cell viability and decreasing cytotoxicity and caspase-^3/7^ activation. (**B**) The ApoTox-Glo^TM^ assay in primary hippocampal neuron cultures from GD 17.5 rat fetuses. Cell viability was decreased but cytotoxicity and caspase-^3/7^ activation were increased after Aβ_1-42_ (5 μM) treatment compared with the control treatment. Treatment with osmotin at three different concentrations (0.1, 0.2, or 0.4 μM) significantly reduced the effects of Aβ_1-42_, thereby increasing cell viability and decreasing cytotoxicity and caspase-^3/7^ activation. #significantly different from the control; *significantly different from the Aβ_1-42_-treated mice. Ns = not significant compared with the Aβ_1-42_-treated mice. n = 3 per experiment.

To measure cell viability/cytotoxicity and apoptosis (using the apoptotic marker caspase-^3/7^), we performed an ApoTox-Glo^TM^ Triplex assay on neuronal HT22 cells and primary cultures of hippocampal neurons from gestational day (GD) 17.5 rat fetuses. Treatment with Aβ_1-42_ (5 μM) reduced the viability of HT22 cells and primary hippocampal neurons and increased the cytotoxicity and activation of caspase-^3/7^ compared with the control treatment. Treatment with osmotin at three different concentrations (0.1, 0.2, and 0.4 μM) significantly reduced the effects of Aβ_1-42_ (5 μM), thereby increasing cell viability and decreasing cytotoxicity and caspase-^3/7^ activation (p < 0.05, Fig. 8A,B), indicating that osmotin reduced Aβ_1-42_ (5 μM)-induced neurotoxicity *in vitro*.

We also determined the toxicity profile of 100% dimethyl sulfoxide (DMSO) in both primary hippocampal neurons and HT22 cells via ApoTox-Glo^TM^ assay. The neurons exposed to the 100% DMSO showed decreased viability as well as increased cytotoxicity and caspase-^3/7^ activation compared with the non-exposed DMSO in both the primary hippocampal and HT22 neuronal cells (Supp. Fig. 2A and B).

## Discussion

The present study is the first to provide evidence that osmotin attenuates Aβ_1-42_-induced memory impairment, synaptic deficits, tau hyperphosphorylation and hippocampal neuronal degeneration in a mouse Aβ_1-42_ model in both the short- and long-term (3 and 40 days post-Aβ_1-42_ injection, respectively). A single injection of Aβ_1-42_ induced memory impairment after both 3 and 40 days; however, the magnitude of the long-term effect was greater than that of the short-term effect, potentially reflecting hippocampal neurodegeneration, tau hyperphosphorylation and, particularly, synaptic degeneration, which is an important characteristic of the early stages of AD. The intracerebroventricular Aβ injection model is a useful complement to transgenic mouse models^34^ for the development and evaluation of therapeutic approaches to AD pathology because the mechanisms underlying many characteristics of AD, including the induction of tau phosphorylation, synaptotoxicity, apoptosis and neurodegeneration, remain elusive. Moreover, the intracerebroventricular Aβ-injection model facilitates behavioral studies in a relatively short timeframe.

Our experimental paradigm (based on the effects of Aβ_1-42_ injection after 3 and 40 days) produced a significant reduction in the percentage of spontaneous alternation behavior, which is associated with hippocampal function^35^. Long-term (40 days) Aβ_1-42_ treatment resulted in a more deleterious effect: the percentage of spontaneous alternation behavior following long-term Aβ_1-42_ treatment was further reduced compared with short-term (3 days) Aβ_1-42_ treatment. Here, we demonstrated that osmotin treatment (15 μg/g, i.p, 4 hr) ameliorates the effects of Aβ_1-42_ on spontaneous alternation behavior, indicating a reduction the degree of spatial memory impairment. Thus, we suggest that the observed improvement in spontaneous alternation behavior due to osmotin treatment demonstrates the neuroprotective effect of osmotin against Aβ_1-42_-induced hippocampal degeneration.

Synaptophysin and SNAP-25 levels are decreased in the brain of AD patients and Aβ-induced rat/mouse models^36^,^37^. Our experimental results revealed that Aβ_1-42_-injection significantly reduced the levels of synaptophysin, SNAP-25, PSD-95 and p-GluR1 at Ser 845 in the mouse hippocampus. The magnitude of the decreases in the levels of synaptophysin, SNAP-25, PSD-95 and p-GluR1 (Ser845) and, consequently, in the percentage of spontaneous alternation behavior in the Aβ_1-42_-treated mice were greater after 40 days than after 3 days. This result suggests a correlation in which mice exposed to long-term Aβ_1-42_ treatment exhibited more deleterious effects and less synaptophysin, SNAP-25, PSD-95 and p-GluR1 (Ser845) expression than mice exposed to short-term Aβ_1-42_ treatment. This observation is consistent with the results of previous studies suggesting that acute Aβ_1-42_ treatment may not exert detrimental effects on presynaptic protein expression^38^,^39^. Aβ-induced synaptotoxicity may be critical in inducing memory dysfunction; reduced synaptophysin expression in the hippocampus is associated with cognitive dysfunction and memory loss in AD patients^40^. The synaptophysin, SNAP-25, PSD-95 and p-GluR1 (which is associated with spatial memory) in the Aβ_1-42_-treated mice were protected by osmotin administration, thereby suggesting that protecting pre-and post-synaptic protein markers improves spatial memory.

Aβ accumulation in the human brain has been implicated in neuronal loss and cognitive dysfunction during AD progression^41^. BACE-1 is the key enzyme that initiates Aβ accumulation, and the activity of BACE-1 is the rate-limiting step in APP processing to generate Aβ^42^. BACE-1 expression is increased in response to a variety of events, including hypoxia^43^ and oxidative stress conditions^44^. Interestingly, recent studies have shown that BACE-1 expression is up-regulated by Aβ_1-42_^45^. The results of the present study consistently showed increased levels of BACE-1 expression in the Aβ_1-42_-treated mice; however, BACE-1 was more strongly expressed at 3 days than at 40 days after Aβ_1-42_ treatment, suggesting that either BACE-1 is expressed independently of Aβ_1-42_ or that BACE-1 is involved in a potential negative feedback mechanism . The total Aβ level and Aβ_1-42_-induced BACE-1 expression at both 3 and 40 days after Aβ_1-42_ injection were attenuated by osmotin treatment (Fig. 3A,B).

Hyperphosphorylated tau is the primary component of neurofibrillary tangles. Aβ accumulation precedes the accumulation of hyperphosphorylated tau in the AD brain. Recent studies showed that soluble Aβ oligomers either generated from synthetic Aβ peptides or extracted from the brain of AD patients promote tau phosphorylation^46^,^47^. Previous studies also demonstrated the Aβ-induced hyperphosphorylation of the tau protein via the activation of various kinases, such as GSK-3β, mitogen activated protein (MAP) kinase and cyclin-dependent kinase-5. GSK-3β activation is elevated by Aβ accumulation in primary cultured neurons^48^. Akt, a serine/threonine kinase and an upstream regulator of GSK-3β, prevents GSK-3β activity via the phosphorylation of GSK-3β at Ser 9^49^. In hippocampal sections from APP/PS1 mice, Akt activity was inhibited based on its reduced phosphorylation at Ser473, and this reduced level of p-Akt (Ser 473) was associated with a reduction in the Akt-mediated phosphorylation of GSK-3β at Ser 9 (thereby increasing the activity of GSK-3β)^50^. Several studies established that GSK-3β mediates the hyperphosphorylation of the tau protein *in vivo*^51^. Importantly, the Aβ_1-42_-induced decrease in Akt phosphorylation was ameliorated by osmotin treatment, consistent with the osmotin-mediated reduction in GSK-3β activity (due to the increased phosphorylation of GSK-3β at Ser9), which decreased the hyperphosphorylation of the tau protein. These results showed that osmotin alleviates the hyperphosphorylation of the tau protein via the regulation of the aberrant PI3K/Akt/GSK-3β signaling pathway at both 3 and 40 days after Aβ_1-42_ injection (Figs 4A,B and 5A,B). Thus, the mechanism by which osmotin attenuates the Aβ_1-42_-induced hyperphosphorylation of the tau protein may involve the PI3K/Akt/GSK-3β signaling pathway.

High levels of p53 expression have been observed in the brains of sporadic AD patients and transgenic mouse models carrying mutant familial AD genes^52^. Aβ_1-42_ has been shown to activate caspases accompanied by p53 activation. Caspase activation in response to Aβ injection has been implicated in the biochemical cascade during the final stage of apoptosis^53^. Activated caspase-3 cleaves PARP-1, resulting in apoptosis and neurodegeneration; the hyperactivation of PARP-1 is involved in NAD^+^ depletion, which leads to neuronal death^54^. Thus, our results showed that osmotin suppressed pro-apoptotic p53 expression, thereby reducing the expression of activated caspases and preventing PARP-1 cleavage, indicating that osmotin prevents Aβ_1-42_-induced neuronal apoptosis at both 3 and 40 days after Aβ_1-42_-injection. Additionally, the results of our histomorphological analysis were consistent with our western blot results, confirming that osmotin attenuates Aβ_1-42_-induced neurodegeneration (Figs 6B and 7). Furthermore, we quantified the effect of osmotin against Aβ_1-42_-induced neurodegeneration *in vitro*. Osmotin prevented Aβ_1-42_-induced neurotoxicity in neuronal HT22 cells and primary hippocampal neuronal cultures (Fig. 8A,B).

In conclusion, the results of the present study demonstrated that osmotin reduces Aβ accumulation, BACE-1 expression, synaptotoxicity and memory impairment in an Aβ_1-42_-injected mouse model. We also showed that osmotin alleviates the hyperphosphorylation of the tau protein possibly through the regulation of the PI3K/Akt/GSK-3β signaling pathway. Moreover, osmotin prevents Aβ_1-42_-induced apoptosis and neurodegeneration via the suppression of p53 expression, thereby reducing caspase-9 and capase-3 activation and PARP-1 cleavage.

Adiponectin and its receptors have been associated with various metabolic diseases, including diabetes, obesity, and cardiovascular and neurodegenerative diseases^55^. Moreover, because osmotin is a homolog of adiponectin, osmotin may act via the adiponectin receptor. The adiponectin receptor regulates AD-associated pathways such as lipid oxidation, glucose uptake and insulin signaling^56^. Adiponectin is a pleotropic endogenous adipokine that displays anti-inflammatory and protective activities in various metabolic disorders^57^. Osmotin, a homolog of adiponectin, is a natural, easily accessible and acidically stable protein that is ubiquitously expressed in edible fruits and vegetables. We suggest that osmotin represents a novel potential candidate agent for the treatment of various chronic and metabolic diseases, including neurodegenerative diseases such as AD; however, further mechanistic studies are needed to confirm this.

## Materials and methods

### Materials

Aβ _1-42_ peptides were purchased from Sigma Chemical Co. (St. Louis, MO, USA). Osmotin purified with some modification as previously described^58^. The detailed osmotin extraction and purification procedure is described in the supplementary information.

### Animals

Male wild type C57BL/6J mice (25–30 g, 8 weeks old) were purchased from Jackson Laboratory (Bar Harbor, ME, U.S.A). The mice were acclimatized for 1 week in the university animal house under a 12-h/12-h light/dark cycle at 23 °C with 60 ± 10% humidity and provided with food and water ad libitum. The mice maintenance and treatment were carried out in accordance with the animal ethics committee (IACUC) guidelines issued by the Division of Applied Life Sciences, Department of Biology at Gyeongsang National University, South Korea. All efforts were made to minimize the number of mice used and their suffering. The experimental methods with mice were carried out in accordance with the approved guidelines (Approval ID: 125) and all experimental protocol were approved by the animal ethics committee (IACUC) of the Division of Applied Life Sciences, Department of Biology at Gyeongsang National University, South Korea.

### Drug treatment

Human Aβ _1-42_ peptide was prepared as a stock solution at a concentration of 1 mg/ml in sterile saline solution, followed by aggregation via incubation at 37 °C for 4 days. The aggregated Aβ_1-42_ peptide or vehicle (0.9% NaCl, 3 μl/5 min/mouse) was stereotaxically administered intracerebroventricularly using a Hamilton microsyringe (−0.2 mm anteroposterior (AP), 1 mm mediolateral (ML) and −2.4 mm dorsoventral (DV) to the bregma) under anesthesia in combination with 0.05 ml/100 g body weight Rompun (Xylazine) and 0.1 ml/100 g body weight Zoletil (Ketamine). We performed the stereotaxic surgical procedure in a separate heated room in which the heating system was designed to control the body temperature (maintained at 36 °C–37 °C). The temperature was monitored regularly using a thermometer because anesthesia decreased the body temperature of the animals and thus induced tau phosphorylation^59^.

We optimized the dose of osmotin according to our preliminary studies. A single dose of 15 μg/g osmotin (dissolved in 0.9% NaCl saline) was administered intraperitoneally (i.p.) at 3 and 40 days following Aβ_1-42_ injection. The control mice received an equal volume of 0.9% NaCl saline i.p. at 3 and 40 days post-injection with 0.9% NaCl.

### Spontaneous alternation in a Y-maze test

In the 3 and 40 days post-injection Aβ_1-42_ mice, the Y-maze test was performed at 4 hr following osmotin and saline administration (n = 15/group). The Y-maze was constructed of black-painted wood. Each arm of the maze was 50 cm long, 20 cm high and 10 cm wide at the bottom and the top. Each mouse was placed in the center of the apparatus and was allowed to move freely through the maze for three 8-min sessions. The series of arm entries was visually observed. Spontaneous alternation was defined as the successive entry into each of the three arms by the mice. Alternation behavior (%) was calculated as [successive triplet sets (consecutive entry into the three different arms)/total number of arm entries-2] x 100.

### Protein extraction from mouse brain

After behavioral analysis in the 3 and 40 days post-injection Aβ_1-42_ mice, the mice were killed without anesthesia. The brains were immediately removed and hippocampus was dissected carefully and the tissues were frozen on dry ice and stored at −80 °C. The hippocampal tissue were homogenised in 0.01 M phosphate buffered saline (PBS) with phosphase inhibitor and protease inhibitor cocktail. The samples were then centrifuged at 10,000 Xg at 4 °C for 25 minutes. The supernatants were collected and stored at −80 °C.

### Western blot analysis

The protein concentration was measured (BioRad protein assay kit, Bio-Rad Laboratories, CA, USA). Equal amounts of protein (20–30 μg) were electrophoresed under the same experimental conditions using 4–12% Bolt^TM^ Mini Gels and MES SDS running buffer 1x (Novex, Life Technologies, Kiryat Shmona, Israel) with broad-range prestained protein marker (GangNam stain^TM^, Intron Biotechnology) as a molecular size control. The membranes were blocked in 5% (w/v) skim milk to reduce non-specific binding and incubated with primary antibodies overnight at 4 °C at a 1:1,000 dilution. After reaction with a horseradish peroxidase-conjugated secondary antibody, as appropriate, the proteins were detected using an ECL detection reagent according to the manufacturer’s instructions (Amersham Pharmacia Biotech, Uppsala, Sweden). The X-ray films were scanned, and the optical densities of the bands were analyzed via densitometry using the computer-based Sigma Gel program version 1.0 (SPSS, Chicago, IL, USA).

### Antibodies

The following primary antibodies were used in the western blot analysis. Rabbit-anti-synaptophysin, anti-caspase-3, anti-cleaved casapse-3, anti-phospho-α-amino-3-hydroxy-5-methyl-4-isoxazolepropionic acid receptors) AMPAR1s (p-GluR1) Ser845), anti-PSD-95, anti-p-PI3K (Y458/Y199), total anti-PI3K, anti-p-Akt (Ser473), anti-total Akt, anti-caspase-9, anti-total tau, and anti-β-actin from Cell Signaling Technology, Beverly, MA,USA. The mouse-anti-Aβ (D-11), rabbit-anti-BACE-1, goat-anti-SNAP25, goat-anti-pGSK3 β(Ser9), rabbit-anti-total GSK3β, mouse-anti-total GluR1, rabbit-anti-p-Tau (Ser 413), mouse-anti-p53, mouse-anti-poly (ADP-ribose) polymerase-1(PARP-1) from Santa Cruz, Biotechnology, CA,USA.

### Tissue collection and sample preparation

After Y-maze analysis, the experimental mice of the 40 days group (n = 5 mice per group) were transcardially perfused with 4% ice-cold paraformaldehyde, and the brains were post-fixed for 72 hr in 4% paraformaldehyde and transferred to 20% sucrose for 72 hr. The brains were frozen in O.C.T compound (A.O, USA), and 14-μm coronal sections were cut using a CM 3050C cryostat (Leica, Germany). The sections were thaw-mounted on ProbeOn Plus charged slides (Fisher, USA).

### Thioflavin S staining

The sections were washed twice for 10 minutes in 0.01 M PBS, then immersed in a Coplin jar containing fresh 1% thioflavin S (Sigma Chemical Co., St. Louis, MO, USA), and stained at room temperature for 10 min. Sections were incubated into 70% ethanol for 5 min, rinsed 2 times in water, and glass coverslips were mounted with propidium iodide (PI) (Invitrogen, Carlsbad, CA, USA). Strong green fluorescence of thioflavin S was observed on confocal laser-scanning microscope. For quantitative analysis, a percentage of plaque area/number of plaques was calculated by using the ImageJ analysis program.

### Single and double immunofluorescence

The slides were washed twice for 15 minutes in 0.01 M PBS, followed by blocking for 1 hr in 5% normal goat or bovine serum. After blocking, the slides were incubated overnight in mouse anti-Aβ (6E10) antibody (Covance, 5858 Horton Street, Suite 500, California USA) rabbit anti-synaptophysin (Cell Signaling Technology, Beverly, MA, USA) and anti-p-tau (Ser413) (Santa Cruz, Biotechnology, CA, USA) antibodies diluted 1:100 in blocking solution. Following, incubation in the primary antibodies, the sections were incubated for 1.5 hr in FITC bovine anti-mouse / TRITC-labelled goat-anti rabbit antibodies (1:50) (Santa Cruz Biotechnology, CA, USA). In case of double immunofluorescence subsequently, after incubation in the goat-anti rabbit TRITC-labelled antibody, the sections were incubated overnight in mouse anti-Aβ (D-11) (Santa Cruz, Biotechnology, CA, USA) (1:100), followed by incubation in the FITC-labelled rabbit anti-mouse antibody (1:50) (Santa Cruz Biotechnology, CA, USA) for 1.5 hr under the same conditions. After incubation in this secondary antibody, the slides were washed with PBS, and the slides were mounted with 4′, 6′-diamidino-2-phenylindole (DAPI) and Prolong Antifade Reagent (Molecular Probe, Eugene, OR, USA). The synaptophysin and p-Tau (Ser413) (both red), Aβ (6E10) and Aβ (D-11) (both green); and DAPI (blue) staining patterns were examined using a confocal laser-scanning microscope (Flouview FV 1000, Olympus, Japan).

### Immunohistochemistry

The slides were washed twice for 15 minutes in 0.01 M PBS, followed by quenching for 10 minutes in a solution of methanol containing 30% hydrogen peroxidase and incubated for 1 h in blocking solution containing 5% normal goat serum and 0.3% Triton X-100 in PBS. After blocking, the slides were incubated overnight in rabbit anti-caspase-3 antibody (Cell Signaling Technology, Beverly, MA, USA) and goat anti-p-GSK3 β (Ser9) diluted 1:100 in blocking solution. Following incubation with primary antibody, the sections were incubated for 1 h in biotinylated goat anti-rabbit and rabbit anti-goat secondary antibody diluted 1:500 in PBS and subsequently incubated with ABC reagents (Standard VECTASTAIN ABC Elite Kit; Vector Laboratories, Burlingame, CA) for 1 h in the dark at room temperature. The sections were washed twice with PBS and incubated in 3, 3′-diaminobenzidine tetra hydrochloride (DAB). The sections were washed with distilled water, dehydrated in graded ethanol (70%, 95% and 100%), placed in xylene and coverslipped using mounting medium. The active caspase-3 and phospho-GSK3β (Ser9)-positive cells in the DG, CA1 and CA3 regions of the hippocampus were analyzed using the ImageJ analysis program.

### Nissl staining

The sections were washed twice for 15 min in 0.01 M PBS and incubated in 0.5% cresyl violet staining solution (containing few drops of glacial acetic acid) for 10-15 minutes at room temperature. Following incubation the sections were washed with distilled water and dehydrated gradually in ethanol (70%, 95% and 100%). After dehydration placed in xylene and coverslipped using non-fluorescence mounting medium. The cells in the CA1, CA3 and DG regions of the hippocampus were analyzed using the ImageJ analysis program.

### Aβ_1-42_ oligomer preparation for *in vitro*

The Aβ_1-42_ peptide (Sigma Chemical Co., St. Louis, MO,USA) was initially dissolved in 100% hexafluoroisopropanol (HFIP). After evaporation of HFIP under vacuum, the peptides were reconstituted in dimethyl sulfoxide (DMSO) to generate a suspension of 5 mM. This 5 mM solution was further diluted to 100 μM in F12 medium (Gibco by life technologies, Grand Island, NY, USA) lacking phenol red. This was incubated at 5 °C for 24 hr. The peptide solution was then centrifuged at 14,000 rpm at 4 °C for 10 min. The supernatant was collected as the oligomeric (monomeric, dimeric and trimeric) Aβ peptide, as confirmed via SDS-PAGE.

### ApoTox-Glo^TM^ Triplex assay

ApoTox-Glo^TM^ Triplex Assay (Promega Corporation, 2800 Woods Hollow Road Madison, WI53711-5399, USA) was performed to assess viability, cytotoxicity and caspase-^3/7^ activation within a single 96 well assay. The first part of the assay simultaneously measures two protease activities as markers of cell viability and cytotoxicity.

Cultures of primary hippocampal neurons from gestational day (GD) 17.5 rat fetuses (2 × 10^4^ cells) were prepared with some modification as we previously described^60^. Mouse hippocampal neuronal HT22 cells (2×10^4^), a generous gift from Prof. Koh (Gyeongsang National University, S.Korea) were cultured in Dulbecco’s modified Eagle’s medium (DMEM) (Gibco by life technologies, Grand Island, NY,USA) supplemented with 10% fetal bovine serum (FBS) and 1% antibiotics at 37°C in humidified air containing 5% CO2. For preparation of Aβ_1-42_, osmotin and vehicle exposure, the cells were transferred to the 35 mm Petri dishes (Nunc A/S, Kamstrupvej 90.P.O.Box 280 DK-4000 Rosklide, Danmark) and used at 70% confluences. On the experiment day, the cells were treated with Aβ_1-42_ (5 μM) and osmotin at final concentrations of 0.1, 0.2 and 0.4 μM for 24 h, except the control group.

Moreover to assess the 100% DMSO toxicity we treated both primary hippocampal neurons and HT22 cells (2×10^4)^ with 100% DMSO or 1x PBS for the control for 1 hr at 37 °C in humidified air containing 5% CO2.

For the assay, 20 μl of the viability/cytotoxicity reagent containing both GF-AFC substrate and bis-AAF-R110 substrate was added to all of the wells and briefly mixed using orbital shaking (500 rpm for 30 seconds) and incubated for 1 hr at 37 °C. The fluorescence was measured at two wavelengths: 400_Ex_/505_Em_ (viability) and 485_Ex_/520_Em_ (cytotoxicity).

The GF-AFC substrate enters live cells and is cleaved by a live-cell protease to release AFC. The bis-AAF-R110 substrate does not enter live cells but rather is cleaved by a dead-cell protease to release R110.

The live-cell protease activity is restricted to intact viable cells and measured using a fluorogenic, cell-permeant, peptide substrate (glycyl-phenylalnyl-aminofluorocoumarin, GF-AFC). A second fluorogenic, cell-impairment peptide substrate (bis-alanylalanyl-phenylalanyl-rhodamine 110; bis-AAF-R110) was used to measure dead-cell protease activity released from cells that have lost membrane integrity.

The second part of the assay uses a luminogenic caspase^3/7^ substrate, containing the tetrapeptide sequence DEVD, in a reagent to measure caspases activity. The caspase-Glo reagent was added (100 μl) to all of the wells and briefly mixed using orbital shaking (500 rpm for 30 seconds). After incubation for 1 h at room temperature, the luminescence was measured to determine caspases^3/7^ activation.

### Statistical analysis

The western blot bands were scanned and analyzed through densitometry using the Sigma Gel System (SPSS Inc., Chicago, IL). The density values were expressed as the means ± standard error mean (SEM). The Image-J software was used for immunohistological quantitative analysis. One-way analysis of variance (ANOVA) followed by a two-tailed independent Student’s *t-test* was used for comparisons among the treated groups and the control. The calculations and graphs were made through Prism 5 software (Graph-Pad Software, In., San Diego, CA). P values less than 0.05 were considered to be statistically significant. **#**indicates significantly different from the vehicle treated control group while *indicates significantly different from the Aβ_1-42_-treated groups. *p < 0.05, **p < 0.01 and ***p < 0.001; and **#**p < 0.05, ##p < 0.01 and ###p < 0.001.

## Additional Information

**How to cite this article**: Ali, T. *et al.* Osmotin attenuates amyloid beta-induced memory impairment, tau phosphorylation and neurodegeneration in the mouse hippocampus. *Sci. Rep.* **5**, 11708; doi: 10.1038/srep11708 (2015).

## Supplementary Material

Supplementary Information
