# Supplementary material for: Osmotin attenuates amyloid beta-induced memory impairment, tau phosphorylation and neurodegeneration in the mouse hippocampus
Source: Sci Rep. 2015 Jun 29;5:11708. doi: 10.1038/srep11708 (PMC4484370; doi:10.1038/srep11708)
Supplement: Supplementary Information [file srep11708-s1.pdf]

ORIGINAL RESEARCH ARTICLE

Osmotin attenuates amyloid beta-induced memory impairment, tau phosphorylation and neurodegeneration in the mouse hippocampus

Tahir Ali, Gwang Ho Yoon, Shahid Ali Shah, Hae Young Lee and Myeong Ok Kim[1]*

Department of Biology and Applied Life Science (BK 21), College of Natural Sciences, Gyeongsang National University, Jinju, 660-701, Republic of Korea.

*Corresponding author

Myeong Ok Kim, Prof. Ph.D.

Head of Neuroscience Pioneer Research Center,

Department of Biology and Applied of Life Science, College of Natural Sciences,

Gyeongsang National University, Jinju, 660-701, South Korea

Tel.: +82-55-772-1345 Fax: +82-55-772-1349

E-mail:mokim@gsnu.ac.kr

Extraction and Purification of Osmotin

Material and Methods

Materials

The syringe filters, CM-Sephadex C-25, methanol, ammonium sulphate, sodium chloride, yeast extract, bacto-peptone, dextrose and endotoxin-free water were purchased from Sigma. Endoclear red medium (Endotrap Red 5/1 + mini LAL) was purchased from Hycult Biotech (600 West Germantown Pike Suite 110 Plymouth Meeting, PA 19462 USA). The ultracentrifuge filter tubes, the BCA protein assay and Miracloth were purchased from Fisher Scientific (81 Wyman Street Waltham, MA 02451, USA). Osmotin-compatible dialysis tubing and analytical filters were purchased from VWR (800 East Fabyan Parkway Batavia, Chicago, IL 60510 USA).

Procedure

Osmotin was purified as previously described with some modifications[58].The 25 g/L (428 mM) NaCl-adapted tobacco cells (*Nicotiana tabacum* L. var Wisconsin-38), a gift from Prof. M.L. Narsimhan (Purdue University, West Lafayette, IN 47907-2010,U.S.A), were used for the purification of osmotin. The cells were homogenised in lysis buffer (20 mM potassium phosphate [pH 6.0], 1 M sucrose, 5 mM EDTA) using a tissue homogeniser on ice ten times for 1 min each. The homogenised cells were centrifuged at 4000 rpm for 15 min at 4 ° C, and the supernatant was collected as an 80% ammonium sulphate precipitation via centrifugation at 4000 rpm for 15 min at 4 °C. Then, the pellets were resuspended in lysis buffer and were incubated overnight for resuspension; this process was repeated at least three times. Then, 1.6 volumes of ice-cold methanol (AR grade) was added to each fraction of the ammonium

sulphate-concentrated protein solution. After incubating methanol supernatant in a fume hood overnight to remove most of the methanol, this solution was dialysed against deionised water using osmotin-compatible tubing overnight. Then, the dialysate was resuspended in 20 mM potassium phosphate [pH 6.0] and loaded on an equilibrated CM-Sephadex C-25 column. The column loaded with the dialysate was washed with three bed volumes of washing buffer (20 mM potassium phosphate, pH 6.0) and was slowly treated with elution buffer (20 mM potassium phosphate, 0.5 M sodium chloride). Each fraction that eluted from the column was subjected to SDS-PAGE to ascertain the protein composition of the fractions. Then, the fractions containing the osmotin effluent were centrifuged using ultracentrifuge filter tubes at 5000 rpm for 10 min at 10 °C to obtain crystals of osmotin. The osmotin crystals were resuspended in endotoxin-free water (HPLC grade), and the endotoxin IC_{50} values in yeast (S. cerevisiae BWG1-7a), and quantification was performed according to standard procedures.

We obtained 7.08 mg/ml osmotin in 1/8th strength PBS. The endotoxin activity was identified as an IC_{50} of 4 µg/ml; the endotoxin levels were 0.42EU/ml.

References.

58. Singh, N. K., Bracker, C. A., Hasegawa, P. M. & Handa, A. K. Characterization of osmotin: A thaumatin-like protein associated with osmotic adaptation in plant cells. *Plant Physiol* **85**, 529-536 (1987)

Figure legend.

Supp. Fig.1. Osmotin reduced the Aβ (6E10) immunofluorescence reactivity in the 40 days $A\beta_{1-42}$-treated groups. # significantly different from the vehicle-treated control mice; ∗significantly different from the $A\beta_{1-42}$-treated mice. n=5 mice/group, n=3 experiment. Magnification 40 x. Scale bar = 50 μm.

Supp. Fig.2. Determination of cytotoxicity profile of 100 % DMSO. (A) The histogram results indicated the Apo-Tox Glo^{TM} assay results of 100 % DMSO in HT22 cells (B). The histogram results indicated the Apo-Tox Glo^{TM} assay of 100% DMSO in primary hippocampal culture neurons. # significantly different from the control. n=3 per experiment.

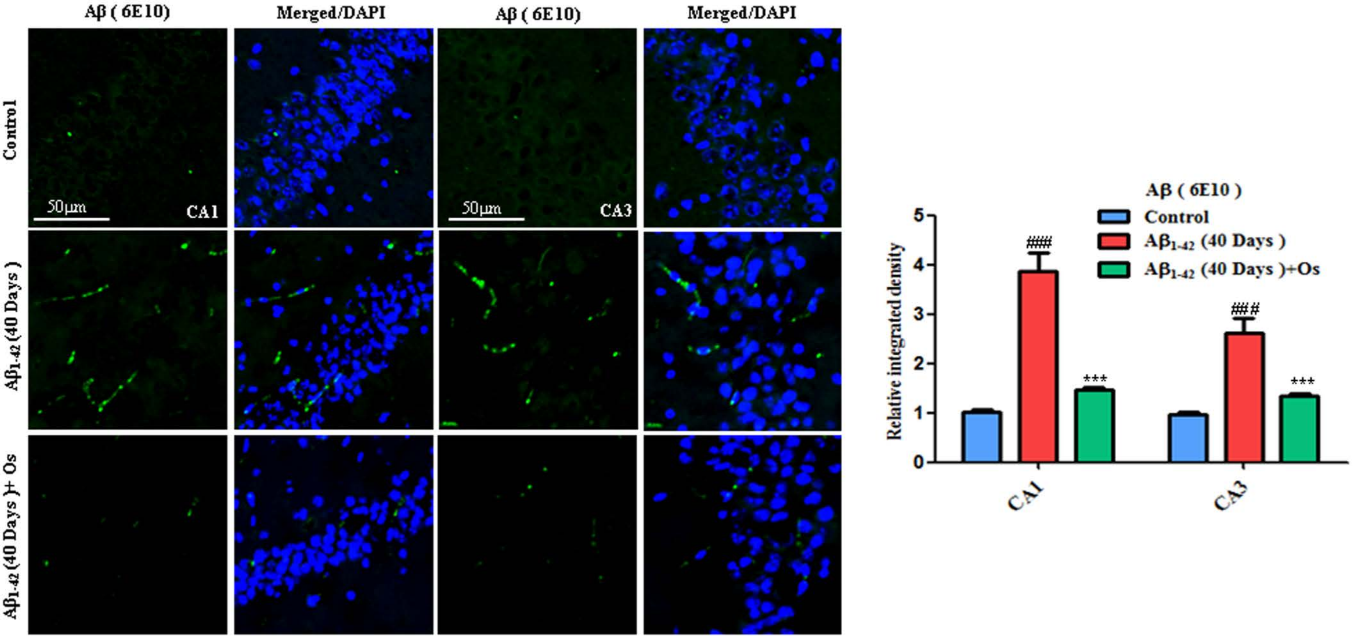

Aβ (6E10)
Merged/DAPI
Aβ (6E10)
Merged/DAPI
Control
Aβ1-42 (40 Days)
Aβ1-42 (40 Days)+ Os
50μm
CA1
50μm
CA3
Aβ (6E10)
Control
Aβ1-42 (40 Days)
Aβ1-42 (40 Days)+Os
Relative integrated density
5
4
3
2
1
0
###
***
###
***
CA1
CA3

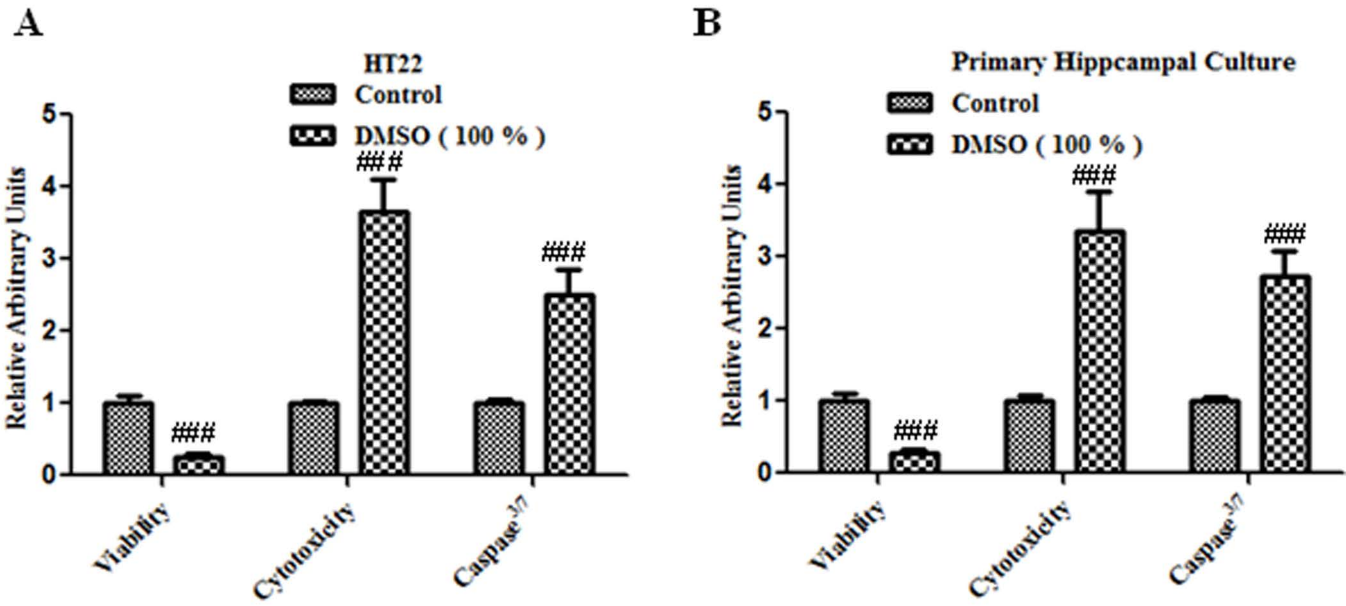
A
HT22
Control
DMSO (100 %)
Relative Arbitrary Units
0
1
2
3
4
5
###
###
###
Viability
Cytotoxicity
Caspase 3/7
B
Primary Hippcampal Culture
Control
DMSO (100 %)
Relative Arbitrary Units
0
1
2
3
4
5
###
###
###
Viability
Cytotoxicity
Caspase 3/7
